# Proteinase K resistant cores of prions and amyloids

**DOI:** 10.1080/19336896.2019.1704612

**Published:** 2019-12-26

**Authors:** Vitaly V. Kushnirov, Alexander A. Dergalev, Alexander I. Alexandrov

**Affiliations:** Research Center of Biotechnology of Russian Academy of Sciences, A.N. Bach Institute of Biochemistry, Moscow, Russia

**Keywords:** Amyloid, prion, prion variants, yeast prions, proteinase K, proteinase resistant core

## Abstract

Amyloids and their infectious subset, prions, represent fibrillary aggregates with regular structure. They are formed by proteins that are soluble in their normal state. In amyloid form, all or part of the polypeptide sequence of the protein is resistant to treatment with proteinase K (PK). Amyloids can have structural variants, which can be distinguished by the patterns of their digestion by PK. In this review, we describe and compare studies of the resistant cores of various amyloids from different organisms. These data provide insight into the fine structure of amyloids and their variants as well as raise interesting questions, such as those concerning the differences between amyloids obtained *ex vivo* and *in vitro*, as well as the manner in which folding of one region of the amyloid can affect other regions.

## Problems in establishing structure of prions and amyloids

Amyloids and their infectious variety, prions, represent fibrils, composed of many molecules of the same protein (protomers), whose altered conformation is reproduced along the fibril. These fibrils are based on intermolecular cross-beta structure, in which beta sheets are parallel to fibril axis and beta strands are perpendicular to it. A unique property of amyloids and prions is that they can have many structural and phenotypic variants (reviewed in [1,2]). For example, as much as 23 different variants were recently reported for the yeast Sup35 prion, and these can be reliably distinguished and faithfully propagated [3].

While the basic structural principle of amyloids, the cross-beta fold, is well known, finer details of their structure remain poorly characterized. This is due to the near impossibility of obtaining 3D amyloid crystals amenable to X-ray structural analysis. Amyloids do not crystallize due to their large size, variable length and propensity for chaotic higher-order aggregation. Nevertheless, recent advances in cryo-electron microscopy and solid-state NMR made possible obtaining structures for some amyloids at near-atomic resolution (reviewed in [4]).

It is important to note that usually only a part of a protomer adopts amyloid structure and forms a stem or core of an amyloid. The rest of a protomer is flexibly attached to the stem [5,6] and if it represents a separate functional domain, it can even retain its original structure and some function [7]. Such modular structure is especially clear in yeast prionogenic proteins, most of which contain separate functional and amyloidogenic domains [8]. Amyloid cores usually display increased resistance to proteases, which allows them to be biochemically separated from other regions of protomers. Then, PK-resistant peptides can be identified by mass-spectrometry or other methods, such as Edman N-terminal sequencing. Of note, some PK-resistant structures may not belong to amyloid core, being intramolecular rather than intermolecular. Further throughout the text, the term core will be used to denote PK-resistant structures of presumably amyloid nature.

Knowledge of the identity of the core is useful for addressing various questions, such as discovery of mutations that interfere with or favour amyloid formation, as well as laborious structural studies, such as solid-state NMR, which are often limited to studying the structure of short proteins. In the frequent case that a single protein produces amyloid variants with different cores, it may be of interest to analyse the correlation of the core size or location with phenotypic manifestations. Finally, data on core sequences can inform algorithms that evaluate the amyloidogenicity of other proteins.

## Proteinase K as a preparative and analytic tool

The most popular enzyme for isolation of amyloid cores is Proteinase K (PK). It is a serine endoprotease that was originally isolated from the fungus *Engyodontium album* (formerly called *Tritirachium album*) [9]. PK is commonly used as a preparative reagent for destruction of proteins, including DNAses and RNAses, in DNA and RNA preparations. Increased resistance to PK proved to be a general property of prions and amyloids and so PK has traditionally been used for their detection and isolation [10–12]. PK digestion revealed that different structural variants of amyloids can differ in size, location and other features, which allowed to distinguish these variants and study the relation between their structure and phenotype.

PK is very tolerant to a range of reaction conditions. It is active over a wide pH range (4–12) [9], while protein denaturants such as guanidine and urea, and strong detergents like SDS or Sarcosyl only enhance the PK activity [13]. However, the key advantage of PK over other proteases in isolation of amyloid cores is its low amino acid specificity, which allows near-complete hydrolysis of non-core regions. Interestingly, data on the specificity of PK are contradictory. A few old publications [14,15] and popular public databases (e.g. https://web.expasy.org/peptide_cutter/) claim that PK cuts solely or highly preferentially C-terminally of aliphatic and hydrophobic residues. However, PK seems to have almost no specificity. In a recent work, we identified about 400 PK digestion fragments of the yeast Sup35 prion, and their ends did not reveal significant residue specificity [16]. Almost all residues were found at the C-termini of these fragments, or preceded the N-termini, with one exception: PK never cut after or before proline. Also, cysteine and tryptophan were not tested since they are absent from the Sup35 prion domain. The PK fragments of *in vitro* assembled amyloids of another yeast prion protein, Ure2, support our observation. PK cut after non-hydrophobic residues of Ure2 (glutamine, asparagine, glycine, serine) no less frequently than after hydrophobic ones [5] and the same was observed for PrP [17]. The difference with the earlier data on PK specificity may be in the nature of the PK substrates: the earlier experiments used short artificial peptides rather than full-length proteins [15].

## PrP^sc^ cores

PrP is a cell membrane protein with a glycosylphosphatidylinositol (GPI) anchor at its C-terminal Serine-231 and one or two glycoside chains at asparagines 181 and 197 [18]. Its normal form, PrP^C^, is readily digested by PK, while in pathogenic structurally altered PrP^Sc^ a larger or smaller part of the polypeptide is resistant to PK. Human prion diseases, related to the PrP protein, can be divided into two major groups with respect to the regions comprising the PK-resistant core. The first group includes Creutzfeldt–Jakob disease (CJD) of any origin (sporadic, familial, iatrogenic and variant CJD), fatal familial insomnia (FFI) and kuru. The second group includes Gerstmann–Sträussler–Scheinker disease (GSS) and variably protease-sensitive prionopathy (VPSPr) [19].

In the first group, digestion of PrP^Sc^ aggregates by PK removes all or a larger part of the N-terminal region containing octapeptide repeats, but leaves the C-terminal region, including the GPI anchor and glycoside chains, intact. This results in the so-called PrP27-30 protein with an apparent molecular mass of 27 to 30 kDa. After removal of glycosides, two types of core fragments become evident, designated as types 1 and 2. Both types have ragged N-termini, i.e. the starting residue is variable. Type 1 core starts near residue 82 and has a molecular mass of 21 kDa; type 2 core starts near residue 97 and has a mass of 19 kDa [20]. Type 1 core was more frequent with methionine at codon 129, and type 2 with valine.

GSS differs from CJD and FFI by slower disease progression and amyloid deposition rather than spongiform change of brain tissue [21]. This difference might be due to a much smaller PrP core of 7–8 kDa observed in GSS, which does not include the C-terminal region, and thus does not include the GPI anchor and glycoside chains. It has ragged N- and C-termini usually starting between residues 70–90 and ending between residues 142–153 [17,22,23]. A larger core of 11 kDa was observed in the Indiana family GSS. In this case, the core started at residue 58 and ended after 150 [24]. In some cases, PrP^Sc^ (GSS) molecules were cross-linked and formed dimers, trimers and tetramers [17].

VPSPr differs from GSS in two respects. First, PrP^Sc^ in VPSPr shows variable and often substantially decreased PK resistance [25,26]. Second, VPSPr occurs without PrP mutations. However, PrP^Sc^ in VPSPr have ~8 kDa PK-resistant core similar to the one in GSS. Thus, VPSPr can be regarded as a sporadic variant of GSS [25]. Interestingly, VPSPr PrP^Sc^ preparations sometimes show fragments of high molecular mass similar to those observed in CJD type 2 [27,28]. This shows possibility of conversion of GSS-type core into CJD-type.

Currently, the model for the spatial structure of PrP^Sc^ (CJD) core with the most experimental support is the four-rung left-handed β-solenoid. This model was first proposed based on electron microscopy data obtained for two-dimensional PrP^Sc^ crystals [29,30]. X-ray fibre diffraction indicated the presence of a repeating unit along the fibre axis, corresponding to four β-strands [31]. Finally, PrP^Sc^ core was reconstructed from cryoelectron microscopy of PK-treated fibres of GPI-anchorless PrP^Sc^ isolated from mice [32]. The diameter of a PrP 27–30 protofilaments was about 4 nm, which allowed to calculate that one PrP molecule occupies about 18 Å along the fibril axis, or four times the interstrand distance in β-sheet. In a good agreement with this, Fourier transform single particle analysis of cryo-EM images of PrP^Sc^ fibres revealed structural repeats of 19.1 Å and ~40 Å[32]. These structures were further refined by molecular dynamics simulations [33]. However, it remains unclear whether other PrP^Sc^ variants, and in particular those related to GSS, reproduce any part of this structure.

## A-synuclein

Α-synuclein is a small natively disordered protein of 140 amino acid residues expressed mainly in neurons. Its amyloid conversion is associated with neurodegenerative diseases called synucleinopathies such as Parkinson’s disease, Lewy body dementia and multiple system atrophy (MSA). The PK-protected core of α-synuclein, recovered from an MSA brain, was shown to be a 7-9kDa peptide that spans residues 31 to 109 [34]. In contrast to α-synuclein, its homologue β-synuclein does not form amyloids. The two proteins differ by the central 12-amino acid peptide, VTGVTAVAQKTV located between positions 71 and 82. This peptide is essential for α-synuclein amyloid formation and was found within the PK-resistant core [35].

Many amyloids formed by different proteins have an ability to seed each other’s polymerization with low efficiency. Self-seeded polymerization *in vitro* of recombinant α-synuclein allowed to obtain two distinct fibrils types, which showed dramatic difference in their ability to cross-seed amyloidization of tau protein, when introduced to either cell culture or mice [36]. PK digestion patterns of these fibrils (termed strain A and strain B) were quite similar with identical sets of five PK-resistant peptides, but differed by their relative intensity. Peptides starting with residues 19–21 dominated in strain A and with 31 – in strain B. Such heterogeneity might reflect the fact that both ‘strain A’ and ‘strain B’ obtained *in vitro* represent a mix of different α-synuclein amyloid conformations, biased towards certain conformational species.

A detailed spatial structure of α-synuclein amyloids was determined by solid-state NMR using *in vitro* generated fibrils, which exhibited pathogenicity in a cell culture test [37]. The α-synuclein amyloid core was formed by the central part of the protein. It had parallel in-register architecture with a novel ‘Greek key’ topology. Cryo-electron microscopy reconstructions confirmed this type of structure, but revealed that α-synuclein fibres are composed of two protofibrils and that the ‘Greek key’ topology is not the only one possible [38–41].

## Het-s structure

The [Het-s] prion of the fungus *Podospora anserina* is involved in self-non-self recognition and a programmed cell death phenomenon called heterokaryon incompatibility [42]. When a [Het-s] prion strain fuses with a *het-S* strain (non-prion alternative allele of the same protein), the resulting cell dies. In the absence of prion, no death reaction occurs. Recombinant HET-s protein with 6-histidine tag at C-terminus (residue 289) polymerized *in vitro* and the obtained fibrils were digested with PK. This resulted in a homogenous peptide of about 7 kDa, which included the C-terminal histidine tag. N-terminal Edman sequencing determined that the peptide starts at lysine-218. The identity of the peptide was further confirmed by its digestion with Lys-C endoproteinase and mass-spectrometry. Thus, the core of the [Het-s] prion spans residues 218–289 and includes the С terminus [43]. Solid-state NMR of the [Het-s] prion core allowed to establish that it represents a left-handed β-solenoid with each molecule making two helical turns [44–46]. It is worth noting that the two turns bear some similarity due to presence of a 21-residue pseudo repeat. This structure includes four β-strands, but almost half of the core is predicted to form loops. Somewhat paradoxically, these loops are resistant to PK, as mentioned above. This allows to suggest that these loops are not exposed, but tightly packed.

## Yeast prions

In contrast to PrP studies, PK has seen only modest use in the mapping of core sequences in yeast prions. These works studied Ure2 [5], Mod5 [47], hybrid *S. cerevisiae-Candida albicans* Sup35 [48] and *S. cerevisiae* Sup35 [16,49]. All these works, except for the last one [16], used *in vitro* generated self-seeded amyloids of respective proteins. Such an *in vitro* approach is not fully correct, because it was never confirmed that the *in vitro* and *in vivo* amyloid structures are equivalent, and it is likely that the self-seeded *in vitro* amyloids are not fully homogenous in their fold. A detailed analysis of discrepancies in results obtained with *in vitro* and *ex-vivo* Sup35 amyloids will be given below.

PK treatment of Ure2p filaments reduces their diameter from 25 nm to 4 nm filaments. Mass spectrometry showed that the latter are composed of prion domain fragments of region 1–70. This region corresponds well to the minimal sequence (1–65) [50] required for propagation of the [*URE3*] prion but is somewhat smaller than the full asparagine and glutamine-rich prion domain 1–89. Study of recombinant Ure2p fibres using hydrogen/deuterium (H/D) exchange revealed that regions protected from exchange are virtually absent [51]. This result contrasts with the reported H/D exchange protected core in Sup35 [52] and looks paradoxical, since formation of any hydrogen bonds should decrease H/D exchange.

Mod5 is the yeast transfer RNA isopentenyltransferase. It is the only yeast prion amyloid protein lacking glutamine/asparagine-rich domains. The [*MOD5*] prion was isolated by its ability to facilitate appearance of an artificial yeast prion Q62-Sup35 [47]. Limited proteolysis of Mod5 amyloids with PK followed by mass spectrometry identified the core of Mod5 as the internal fragment 194–215. Despite the small core size, Mod5 prion aggregates obtained from yeast cells were insoluble in SDS.

A chimeric Sup35 protein was constructed by replacing residues 41–123 of *Saccharomyces cerevisiae* (SC) Sup35 with the corresponding residues 47–141 from *Candida albicans* (CA) [53]. This model protein was designed to bridge the species barrier for Sup35 prion transmission between these yeast species. The chimeric recombinant Sup35 was polymerized *in vitro*, being seeded by amyloids of Sup35 from either SC or CA. In the former case, the PK-resistant core of the obtained amyloids included Sup35 residues 2–45, 49, which roughly corresponds to the SC part. In the latter, two cores coexisted, corresponding to the SC and CA parts (residues 2–45, 49 and 50–128) [48]. Thus, the CA core induced formation of the neighbouring SC core, but not vice versa.

## Yeast Sup35 prion variants

To avoid the uncertainties related to the use of *in vitro*-generated amyloids, we studied Sup35 cores in prion particles isolated from yeast cells [16]. Using limited PK digestion and MALDI-TOF, we mapped Sup35cores for 26 independent [*PSI*^+^] isolates obtained from different sources or induced in different conditions. We observed that the Sup35 core may be composed of up to four separate PK-resistant structures. Such a number of cores is unprecedented. Only one other work described prion or amyloid with more than one PK-resistant element: the above-mentioned hybrid *Saccharomyces-Candida* Sup35 amyloid, which possessed two cores [48].

The wild-type Sup35 PK-resistant structures were located within regions 2–72, 73–124, 125–153, and 154–221, called Regions 1 to 4. These structures, termed Cores 1 to 4, usually occupied a larger part, but not all, of the respective Region. Notably, only one Core was present in all [*PSI*^+^] isolates, the N-terminal Core 1. Besides, very rarely we observed a structure belonging to two of these Regions, located between residues 81 and 144. A similarly located, but slightly larger, structure was observed in *in vitro* Sup35 amyloids [49]. It is important to note that the amyloid nature of structures in Region 4 requires further proof. This region has 21% acidic and 24% basic residues and is not regarded to be amyloidogenic by algorithms [54,55]. On the other hand, the folded state of this region was shown to propagate along Sup35 fibrils [56], which suggests intermolecular interaction and multimolecular structure in this region. Also, NMR indicated that at least some leucine residues of Region 4 are in-register and form hydrogen bonds [57].

Two major PK digestion patterns were observed for the N-terminal Core 1, fully correlating with the [*PSI*^+^] phenotype. In weak [*PSI*^+^] variants the major PK-resistant fragments were 2–42 and 2–45, while in strong variants these were 2–35, 2–38 and 2–70. Thus, [*PSI*^+^] phenotype is defined by the Core 1 structure. It is not clear yet, whether Cores 2 to 4 have any effect on [*PSI*^+^] phenotype.

The Core 1 can be further divided into two parts: region 2–32 (1A) that is fully protected from PK and a partly protected region 33–72 (1B). Almost no resistant peptides fully belonging to the region 1B were observed, which suggests that this region is protected only when connected to region 1A and rapidly degrades when separated from it by PK. Thus, Regions 1A and 1B are likely to be substantially different in their structure. It is possible that the cross-beta structure is restricted to the Region 1A, which is inaccessible for PK, and one can only observe the effects of the region A on the folding and/or accessibility to PK of the region 1B.

Our finding that the fully and partially PK-resistant Sup35 N-terminal regions are of essentially the same size in different [*PSI*^+^] variants somewhat contradicts the different sizes of the region critical for [*PSI*^+^] propagation determined by scanning mutagenesis with proline substitutions and glycine insertions [58]. The critical region spanned residues 5–25 for strong [*PSI*^+^] variant VH, 8–37 for weak [*PSI*^+^] VK and 5–52 for weak [*PSI*^+^] VL [58]. This discrepancy can be explained, at least in part, by the fact that weak [*PSI*^+^] variants are generally less stable and thus should be more sensitive to changes affecting [*PSI*^+^] propagation. Therefore, the apparent critical region can be larger due to the inclusion of less critical residues.

Core 2 was more PK-resistant and more frequently found in weak variants. Usually, it occupied region 91–121 in weak variants and region 82–100 in strong ones, which correlates well with the location of the ‘tail’ region of prion core by Krishnan and Lindquist [59]. Thus, Core 2 parameters somehow depend on Core 1 type. Attempts to selectively eliminate Core 2 by high-level coexpression of Sup35 variant lacking a large part of Core 2 sequence were successful with a strong [*PSI*^+^] but failed with a weak variant. Also of note, in some strong [*PSI*^+^] variants Cores 2 to 4 were prone to change during propagation. All this allows assuming that Core 1 predefines formation of Core 2, and possibly other Cores, but this influence is relatively weak in at least some strong [*PSI*^+^] variants.

Several works indicate that Sup35 prion and its variants have parallel in-register structure. Mass-per-length measurements of the Sup35(1-61)-GFP fibrils obtained *in vitro* by seeding with three different [*PSI*^+^] variants, showed approximately one Sup35 molecule per 0.47 nm [60], which corresponds to the interstrand distance in a β-sheet. Then, it was shown by NMR that tyrosine and phenylalanine residues in the N domain, as well as some leucines in the M domain are located in-register at a distance of about 0.5 nm [57,61,62]. However, Sup35 amyloid that does not have a parallel in-register structure was also reported [63]. A three-rung β-solenoid model was also proposed, based on amino acid proximity studies using fluorescent labels [59]. This model appears unlikely in view of the above data, though it cannot be excluded for uncharacterized [*PSI*^+^] variants.

In the in-register structure, Sup35 occupies one 0.5 nm ‘layer’ along the fibril, but it still remains unclear, how Sup35 folds within this layer. It was proposed that Sup35 is folded into short (6–9 residues) β-strands joined by β-turns and thus forms a serpentine shape [64]. However, our PK digestion data do not support this model, though there is no direct contradiction either. In particular, the serpentine model does not explain or anticipate the described substantial difference between regions 1A and 1B. One cannot exclude a serpentine structure in region 1A, but this requires assuming that this structure is fully resistant to PK. We did not observe any serpentine-like periodicity in PK cutting sites in region 1B and observed increased susceptibility of glutamines at positions 56, 57, 61, 62 and 70–72, which are presumed to be in the middle of a beta strand. On the other hand, some predicted PK cutting sites might be protected due to presence of proline, the only residue not susceptible to PK. The serpentine model also poorly accommodates the observation that two and half of oligopeptide repeats form PK-protected structure, while other repeats do not.

## Comparison of the Sup35 fibrils generated *in vitro* and *in vivo*

Sup35 fibrils spontaneously formed *in vitro* at 4°C yield [*PSI*^+^] mainly of strong type when introduced into yeast cells, and those formed at 37°C produce predominantly weak [*PSI*^+^][65]. Due to this, it was generally assumed that these fibrils adequately reflect the fold of the strong and weak [*PSI*^+^], and a larger part of structural studies of Sup35 amyloid fold was based on this assumption. In particular, Toyama and co-authors showed that the region protected from H/D exchange spans residues 1–37 in strong and 1–70 in weak self-seeded Sup35 fibrils [52]. In line with this, Ohhashi and co-authors observed that major PK-protected peptide in the ‘weak’ fibrils assembled at 37°C is 2–72, but this peptide is absent from digest of the ‘strong’ 4°C assembly [49]. These results, and especially the last one, are opposite to our observations that better protection from PK of the 2–72 fragment is one of the characteristic distinctions of the strong [*PSI*^+^] prions obtained *ex vivo* [16]. Further experiments are required to find out whether this contradiction reflects different properties of *in vitro* fibrils or results from other causes. For now, we can only note that our observations are based on a large set (i.e. 26) of [*PSI*^+^] isolates, where in all cases Sup35 Core 1 digestion pattern corresponded to the [*PSI*^+^] phenotype.

Contradictions between the structures of Sup35 fibrils obtained *in vitro* and *ex vivo* are also suggested by the fact that the presence of cellular components can significantly affect even seeded formation of Sup35 fibrils. Recombinant Sup35NM was seeded by diluted [*PSI*^+^] lysate, and the obtained fibrils were diluted and used to seed Sup35NM in three sequential steps to remove the lysate components. Alternatively, Sup35NM fibrils were assembled directly in a cellular lysate. The Sup35NM fibres in the former case contained 55% of β-sheet and 40% of α-helix, and those assembled in yeast lysates contained 75% of β-sheet and no α-helix [66].

Lastly, *in vitro* generated amyloids can undergo structural rearrangement when introduced into live cells, and recent Sup35 studies provide a vivid example of this. S17R mutation of Sup35 strongly interferes with [*PSI*^+^] propagation in yeast [67]. *In vitro* Sup35NM-S17R formed fibrils with amyloid core (called PrD-C) located between residues 62–144 or 81–148 [49] and thus lacked the N-terminal amyloid core, which was invariably observed in all *ex-vivo* Sup35 prion particles [16]. The PrD-C core could be propagated *in vitro* by wild-type Sup35, but such amyloids showed very low infectivity, when used to transform yeast [*psi*^−^] cells. Furthermore, the obtained transformants were unstable and rapidly converted into a strong suppressor [*PSI*^+^] variant, which was later typed as previously described [58] variant VH [3]. And we showed that VH has an N-terminal core and lacks PrD-C core [16]. Thus, PrD-C core cannot propagate *in vivo* as the only Sup35 prion core, and, apparently, it tends to be lost in the presence of Core 1. However, we sometimes observed a similar, but smaller, core located at residues 91–143 together with the N-terminal Core1 in *ex-vivo* Sup35 prions [16] and our unpublished observations.

## Some similarities of PrP^sc^ and Sup35 prions

Despite many differences between the mammalian PrP^Sc^ and yeast Sup35 prions, some similarities are worth noting. Two very different cores were observed for PrP. The CJD-type cores of about 20 kDa encompass all of the PrP sequence except for the N-terminal repeats and appear to have four-rung beta-helical structure. The GSS type cores of about 7 kDa occupy the region 80–153 and form a currently unknown structure. Yeast Sup35 also can form very different amyloid cores. While *in vivo* the main and often only core is located at the N-terminus, *in vitro* it is possible to obtain amyloids with internal cores at residues 81-144/148 or 61–144. Such a core was obtained with mutant Sup35 (S17R) that is impaired in the formation of the N-terminal core. This resembles the GSS case, in that the GSS core type is related to PrP mutations. The internal core of Sup35 can propagate on wild-type Sup35 *in vitro*, but is rapidly replaced by the N-terminal core in yeast cells. Similarly, the 8-kDa GSS core was not infectious for mice with wild-type PrP, with the exception of few GSS cases with P102L mutation, where PrP^Sc^ was present simultaneously in the 8 kDa and 21 kDa forms [68,69]

It was noted that octapeptide repeats in the N-terminal region of PrP, PHGGGWGQ, are fairly similar to nonapeptide repeats within prion domain of yeast Sup35, PQGGYQQYN [70], and this similarity could be significant. Deletion of one or more repeats from Sup35 interferes with [*PSI*^+^] propagation [71,72], but replacement of the missing repeat with PrP repeat restores the ability to propagate [*PSI*^+^][71]. Mice with deletion of all but one PrP repeats were susceptible to prion infection, but development of disease was slowed by almost two-fold [73]. Some of the Sup35 repeats are sensitive to PK, and some are partially protected, but this protection appears to be due to association with the fully protected core [16]. In the PrP^Sc^, one octarepeat is often protected, and in at least one case four repeats were protected [24].

The reviewed studies demonstrate that numerous aspects of amyloid structures are still unclear, namely: 1) the precise differences between variants and how these differences translate into phenotype; 2) the mechanisms of how folding of one core region affects folding of others; 3) the differences between amyloids obtained *in vitro* and extracted *ex vivo*. The use of PK-resistant core mapping brings a strong, relatively high resolution and medium-throughput tool for the study of these problems. It can also inform the choice of problems which can then be addressed by structural methods with 3D resolution, such as cryoelectron microscopy, NMR and other methods.
